# Multi-modal wound classification using wound image and location by deep neural network

**DOI:** 10.1038/s41598-022-21813-0

**Published:** 2022-11-21

**Authors:** D. M. Anisuzzaman, Yash Patel, Behrouz Rostami, Jeffrey Niezgoda, Sandeep Gopalakrishnan, Zeyun Yu

**Affiliations:** 1 Department of Computer Science, University of Wisconsin-Milwaukee, Milwaukee, WI USA; 2 Department of Electrical Engineering, University of Wisconsin-Milwaukee, Milwaukee, WI 53211 USA; 3 Advancing the Zenith of Healthcare (AZH) Wound and Vascular Center, Milwaukee, WI USA; 4 College of Nursing, University of Wisconsin Milwaukee, Milwaukee, WI USA; 5 Big Data Analytics and Visualization Laboratory, Department of Biomedical Engineering, University of Wisconsin-Milwaukee, 3200 N. Cramer St, EMS E327, Milwaukee, WI 53211 USA

**Keywords:** Computational biology and bioinformatics, Health care, Medical research

## Abstract

Wound classification is an essential step of wound diagnosis. An efficient classifier can assist wound specialists in classifying wound types with less financial and time costs and help them decide on an optimal treatment procedure. This study developed a deep neural network-based multi-modal classifier using wound images and their corresponding locations to categorize them into multiple classes, including diabetic, pressure, surgical, and venous ulcers. A body map was also developed to prepare the location data, which can help wound specialists tag wound locations more efficiently. Three datasets containing images and their corresponding location information were designed with the help of wound specialists. The multi-modal network was developed by concatenating the image-based and location-based classifier outputs with other modifications. The maximum accuracy on mixed-class classifications (containing background and normal skin) varies from 82.48 to 100% in different experiments. The maximum accuracy on wound-class classifications (containing only diabetic, pressure, surgical, and venous) varies from 72.95 to 97.12% in various experiments. The proposed multi-modal network also showed a significant improvement in results from the previous works of literature.

## Introduction

More than 8 million people are suffering from wounds, and the medicare cost related to wound treatments ranged from $28.1 billion to $96.8 billion, according to a 2018 retrospective analysis^1^. This immense number can give us an idea of the population related to wound and their care and management. The most common types of wounds/ulcers are diabetic foot ulcer (DFU), venous leg ulcer (VLU), pressure ulcer (PU), and surgical wound (SW). About 34% of people with diabetes have a lifetime risk of developing a DFU, and more than 50% of diabetic foot ulcers become infected^2^. About 0.15% to 0.3% of people suffer from active VLU worldwide^3^. A pressure ulcer is another significant wound, and 2.5 million people are affected each year^4^. Yearly about 4.5% of people have a surgery that leads to a surgical wound^5^.

The above statistics show that wounds have caused a huge financial burden and may even be life-threatening to patients. An essential part of wound care is to differentiate among different types of wounds (DFU, VLU, PU, SW, etc.) or wound conditions (infection vs. non-infection, ischemia vs. non-ischemic, etc.). To prepare proper medication and treatment guidelines, physicians must first detect the correct wound class. Until the recent advancement of artificial intelligence (AI), wound specialists manually classified wounds. AI can save both time and cost and, in some cases, may give better predictions than humans. In recent years, AI algorithms have evolved into so-called data-driven techniques without human or expert intervention, as compared to the early generations of AI that were rule-based, relying mainly on an expert’s knowledge^6^. This research focuses on wound type classification using a data-driven AI technique named Deep Learning (DL).

Deep learning is prevalent in image processing, with a huge success in medical image analysis. In the general field of image processing and study, some widely used DL algorithms are Convolutional Neural Networks (CNN), Deep Belief Networks (DBN), Deep Boltzmann Machines (DBM), and Stacked (Denoising) Autoencoders^7^. In addition, some of the most common DL methods for medical image analysis include LeNet, AlexNet, VGG 19, GoogleNet, ResNet, FCNN, RNNs, Auto-encoders, Stacked Auto-encoders, Restricted Boltzmann Machines (RBM), Variational Auto-encoders, and Generative Adversarial Networks^8^. Bakator et al.^9^ reviewed CNN, RBM, Self-Advised Support Vector Machine (SA-SVM), Convolutional Recurrent Neural Network (CRNN), DBN, Stacked Denoising Autoencoders (SDAE), Undirected Graph Recursive Neural Networks (UGRNN), U-NET, and Class Structure-Based Deep Convolutional Neural Network (CSDCNN) as deep learning methods in the field of medical diagnosis.

**Figure 1:**
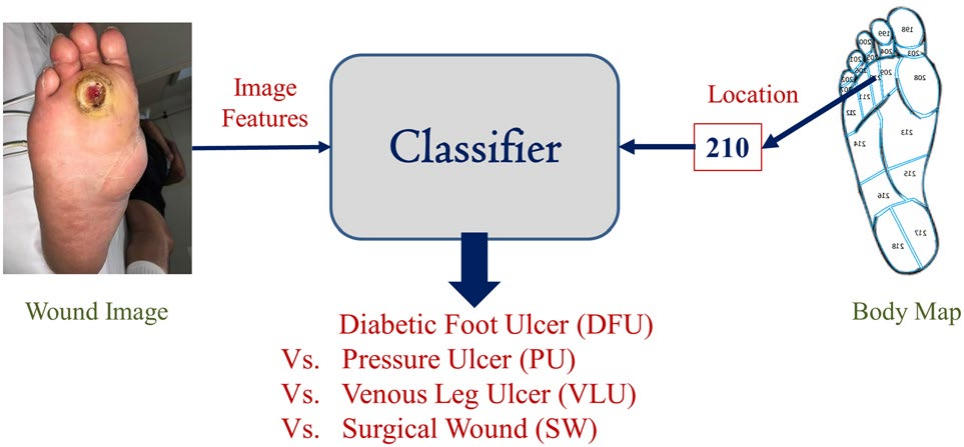
Workflow of this research.

Though there exists some feature-based machine learning and end-to-end deep learning models for image-based wound classification, the classification accuracy is limited due to incomplete information considered in the classifiers. The novelty of the present research is to add wound location as a vital feature to obtain a more accurate classification result. Wound location is a standard entry for electronic health record (EHR) documents, which many wound physicians utilize for wound diagnosis and prognosis. Unfortunately, these locations are documented manually without any specific guidelines, which leads to some inconsistency. In the current work, we developed a body map from which one can select the location of the wound visually and accurately. Then, for each wound image, the wound location was set through the body map, and the location was indexed according to the image file name. Finally, the developed classifier was trained with both image (gained through convolution) and location features and produced superior classification performance compared to image-based wound classifiers. A basic workflow of this research is shown in Fig. 1. The developed wound classifier takes both wound image and location as inputs and outputs the corresponding wound class.

The remainder of the work is organized as follows. Related works on wound classification are discussed in Section “Related works”. Section “Methodology” discusses the methodology, where the dataset, body map, and classification models are described. In Section “Experiment and result and discussion”, experimental setup, results and comparison, and discussion on the results are presented. Finally, the paper is concluded, and some remarks on future directions are given.

## Related works

Wound classification includes wound type classification, wound tissue classification, burn depth classification, etc. Wound type classification considers different types of wounds and non-wounds (normal skin, background, etc.). Background versus DFU, normal skin versus PU, and DFU versus PU are examples of binary wound type classification. In contrast, DFU versus PU versus VLU is an example of multi-class wound type classification. Wound tissue classification differentiates among different types of tissues (granulation, slough, necrosis, etc.) within a specific wound. Burn depth classification measures the depth (superficial dermal, deep dermal, full-thickness, etc.) of the burn wound. As this research focuses on wound type classification, this section discusses existing data-driven wound type classification works. Here, we present machine learning and deep learning-based wound type classification works.

A machine learning approach was proposed by Abubakar et al.^10^ to differentiate burn wounds and pressure ulcers. Features were extracted using pre-trained deep architectures like VGG-face, ResNet101, and ResNet152 from the images and then fed into an SVM classifier to classify the images into burn or pressure wound classes. The dataset used in this study included 29 pressure and 31 burn wound images obtained from the internet and a hospital, respectively. After augmentation, they had three categories: burn, pressure, and healthy skin, with 990 sample images in each class. Several experiments, including binary classification (burn or pressure) and 3-class classification (burn, pressure, and healthy skin), were conducted.

Goyal et al.^11^ used traditional machine learning, deep learning, and ensemble CNN models for binary classification of ischemia versus non-ischemia and infection versus non-infection on DFU images. The authors developed a dataset containing 1459 DFU images that two healthcare professionals labeled. For traditional machine learning, the authors used BayesNet, Random Forest, and Multilayer perceptron. Three CNN networks (InceptionV3, ResNet50, and InceptionResNetV2) were used as deep-learning approaches. The ensemble CNN contained an SVM classifier that takes the bottleneck features of three CNN networks as input. The test evaluation showed that traditional machine learning methods performed the worst, followed by deep-learning networks, while the ensemble CNN performed the best in both binary classifications. The authors reported an accuracy of 90% for ischemia classification and 73% for infection classification.

A novel CNN architecture named DFUNet was developed by Goyal et al.^12^ for binary classification of healthy skin and DFU skin. A dataset of 397 wound images was presented, and data augmentation techniques were applied to increase the number of images. The proposed DFUNet utilized the idea of concatenating the outputs of three parallel convolutional layers with different filter sizes. An accuracy of 92.5% was reported for the proposed method.

A CNN-based method was proposed by Aguirre et al.^13^ for VLU versus non-VLU classification from ulcer images. This study used a pre-trained VGG-19 network to classify the ulcer images in the two categories mentioned. First, a dataset of 300 pictures annotated by a wound specialist was proposed, and data pre-processing and augmentation were conducted before the network training. Then, the VGG-19 network was pre-trained using another dataset of dermoscopic images. The authors reported 85%, 82%, and 75% accuracy, precision, and recall.

Shenoy et al.^14^ proposed a CNN-based method for binary classification of wound images. In this study, they used a dataset of 1335 wound images collected via smartphones and the internet. The authors considered nine different labels (wound, infection (SSI), granulation tissue, fibrinous exudates, open wound, drainage, steri strips, staples, and sutures) for the dataset, where for each label, two subcategories (positive and negative) were considered. The authors used a modified VGG16 network named WoundNet as the classifier, pre-trained using the ImageNet dataset. In addition, the researchers created another network called Deepwound, an ensemble model that averaged the results of three individual models. The reported accuracy varies from 72% (drainage) to 97% (steri strips), where the accuracy for the class “wound” is 82%.

A binary patch classification of normal skin versus abnormal skin (DFU) was performed by Alzubaidi et al.^15^ with a novel deep convolutional neural network named DFU_QUTNet. First, the authors introduced a new dataset of 754-foot images from a diabetic hospital center in Iraq. From these 754 images, 542 normal skin patches and 1067 DFU patches were generated. Then, in the augmentation step, they multiplied the number of training samples by 13, using flipping, rotating, and scaling transformations. The proposed network was a deep architecture with 58 layers, including 17 convolutional layers. The performance of their proposed method was compared with those of other deep CNNs like GoogLeNet, VGG16, and AlexNet. The maximum reported F1-Score was 94.5%, obtained from combining the DFU_QUTNet architecture with SVM.

Rostami et al.^16^ proposed an end-to-end ensemble DCNN-based classifier to classify entire wound images into multiple classes, including surgical, diabetic, and venous ulcers. The output classification scores of two classifiers based on patch-wise and image-wise strategies were fed into a Multi-Layer Perceptron to provide a superior classifier. A new dataset of authentic wound images containing 538 images from four different types of wounds was introduced in this research. The reported maximum and average classification accuracy values were 96.4% and 94.28% for binary and 91.9% and 87.7% for 3-class classification.

Sarp et al.^17^ classified chronic wounds into four classes (diabetic, lymphovascular, pressure injury, and surgical) by using an explainable artificial intelligence (XIA) approach to provide transparency on the neural network. The dataset contained 8690 wound images collected from the data repository of eKare, Inc. Mirroring, rotation, and horizontal flip augmentations were used to increase the number of wound images and to balance the number of pictures in each class. Transfer learning on the VGG16 network was used as the classifier model. The authors reported an average F1 score of 0.76 as the test result. The XIA technique can provide explanation and transparency for the wound image classifier and why the model would think a particular class may be present.

Though some wound type classification works from wound images exist, to the best of our knowledge, there is no automated wound classification work based on the wound location feature. This research is the first work that incorporates wound location for automatic wound type classification and proposes a multi-modal network that uses both wound image features and location features to classify a wound.

## Methodology

### Dataset

In this research, two different datasets were used for our experiments. Our team developed one dataset called AZH Dataset, and the other was a public dataset called Medetec Dataset. We also developed a mixed dataset with the datasets mentioned above named AZHMT Dataset. A brief discussion of these datasets is given below:

#### AZH dataset

AZH dataset was collected over a two-year clinical period at the AZH Wound and Vascular Center in Milwaukee, Wisconsin. The dataset includes 730 wound images in .jpg format. The images are of various sizes, where the width ranging from 320 to 700 pixels and the height ranging from 240 to 525 pixels. These images contain four different wound types: venous, diabetic, pressure, and surgical. iPad Pro (software version 13.4.1) and a Canon SX 620 HS digital camera were used to capture the images, and labeling was done by a wound specialist from the AZH Wound and Vascular Center. For most images in our dataset, each image was taken from a separate patient. But there were a few cases where multiple photos were taken from the same patient at different body sites or various healing stages. For the latter case, the wound shapes were different, so they were considered separate images. Unfortunately, due to the limited data resources, we could not increase the data samples in our dataset. This work did not involve any experiments on humans or the use of human tissue samples. We used wound image data from an external source, which is now publicly available at https://github.com/uwm-bigdata/Multi-modal-wound-classification-using-images-and-locations. All data have been carefully inspected and de-identified. This public dataset contains only wound ROIs (i.e., wounds and surrounding skins) to protect patient identities by removing all unnecessary and personal information from the images. The use of the dataset has been inspected by The University of Wisconsin-Milwaukee to meet the university policy.

#### Medetec dataset

Medetec wound database^18^ contains free stock images of all types of open wounds. We randomly collected 358 images from these three categories: diabetic, pressure, and arterial and venous leg ulcers. The arterial and venous leg ulcer images are not separated in the Medetec database, so we considered them in the same category. This dataset does not contain any surgical wound images. All the images are in .jpg format, where the weight varies from 358 to 560 pixels, and the height varies from 371 to 560 pixels. This external public dataset was used to perform the robustness and reliability testing of the developed model.

#### AZHMT dataset

This dataset is the mixer of all the images from the AZH and Medetec datasets. This dataset contains 1088 wound images in .jpg format. AZHMT includes four wound classes: diabetic, pressure, surgical, and arterial + venous leg ulcers. The width of these images varies from 320 to 700 pixels, and the height ranges from 240 to 560 pixels. AZHMT dataset was created for testing the effect of a bigger dataset on our developed model.

### Body map for location

A body map is a labeled, simplified, and symbolic diagram of the entire body of the person, which should be phenotypically right^19^. Medical practitioners use body maps to locate bruises, wounds, or body breakage on a patient’s body. Moreover, forensic scientists use body diagrams to help them identify and determine body changes during a postmortem examination. Doctors use body maps to analyze the location of a given infection in patients^20^. A detailed body map helps doctors determine which other part of the body to be cautious about during the wound’s rehabilitation process. Moreover, a body map is a piece of medical evidence during a scientific study. A health practitioner can use notable body changes shown by a body map as a backup of an existing ailment affecting the patient internally.

Wound history is another benefit attributed to efficient body mapping. A doctor can collect information on the wound’s cause, previous measures adopted in providing care to the wound, and underlying health complications such as diabetes that would deter the healing process. Detailed wound history needs to be collected and all causes explored to avoid delayed or static healing. Body mapping contributes to wound treatment localization significantly. Pain location, activities of daily living, and the type of wound are factors that a doctor should consider in the localization process. Wilson asserts that a wound in the heel area and a wound on the lower abdomen or joint area would not have a similar rehabilitation technique. The wound on the heel would need the doctor to consider the weight issue instead of the wound on the lower abdomen. Therefore, the doctor would need to localize their examination and the treatment process depending on the wound’s location and other external factors that directly affect the wound weight and joint movement^20^.

**Figure 2:**
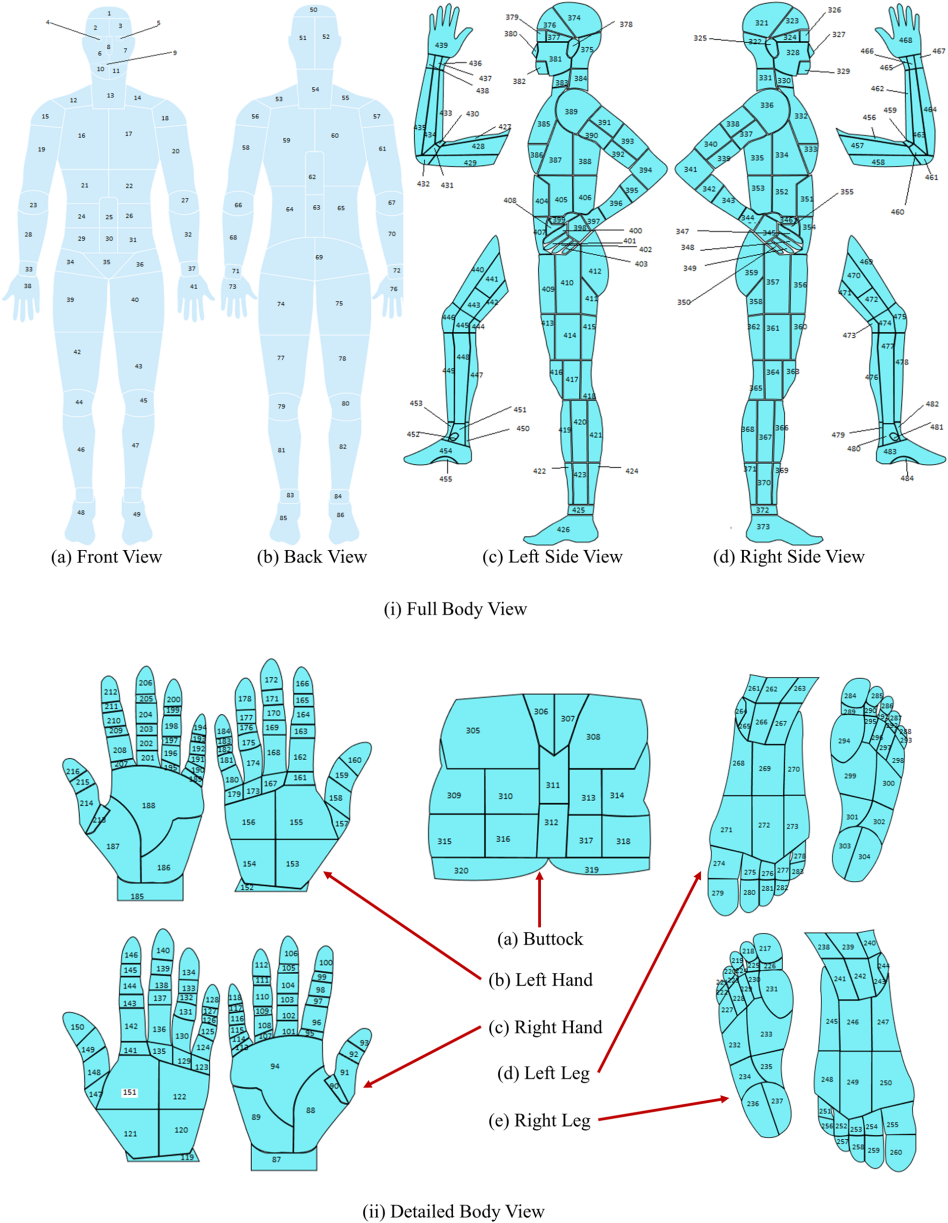
Body map for location selection.

A body map with 484 total parts was designed to avoid the body map’s complexity. The body map was prepared using PaintCode^21^. The initial reference to the body map was obtained from^22–24^. The ground truth diagram for the design is based on the Original Anatomy Mapper^25^. Each label and outline were directly paired with the labeling provided by the anatomy mapper^25^. To avoid the extreme complexity of drawing every detailed feature of the body map, a total of 484 feature or region was pre-selected and approved by wound professionals at the AZH wound and vascular center. The developed body map is shown in Fig. 2. Here each number represents a location. A few examples of the locations and their corresponding numbers are shown in Table 1.

**Table 1:** Examples of locations and their corresponding mapping.

Left hand front		Right leg bottom		Buttock	
Location	Reference number	Location	Reference number	Location	Reference number
Left dorsal wrist	152	Right distal plantar first toe	217	Left posterior lower back	305
Left proximal lateral dorsal hand	153	Right proximal plantar first toe	226	Superior gluteal	311
Left proximal medial dorsal hand	154	Right distal lateral mid plantar foot	232	Inferior gluteal	312
Left distal phlanax of dorsal little finger	184	Right medial heel	237	Left gluteal fold	320

**Figure 3:**
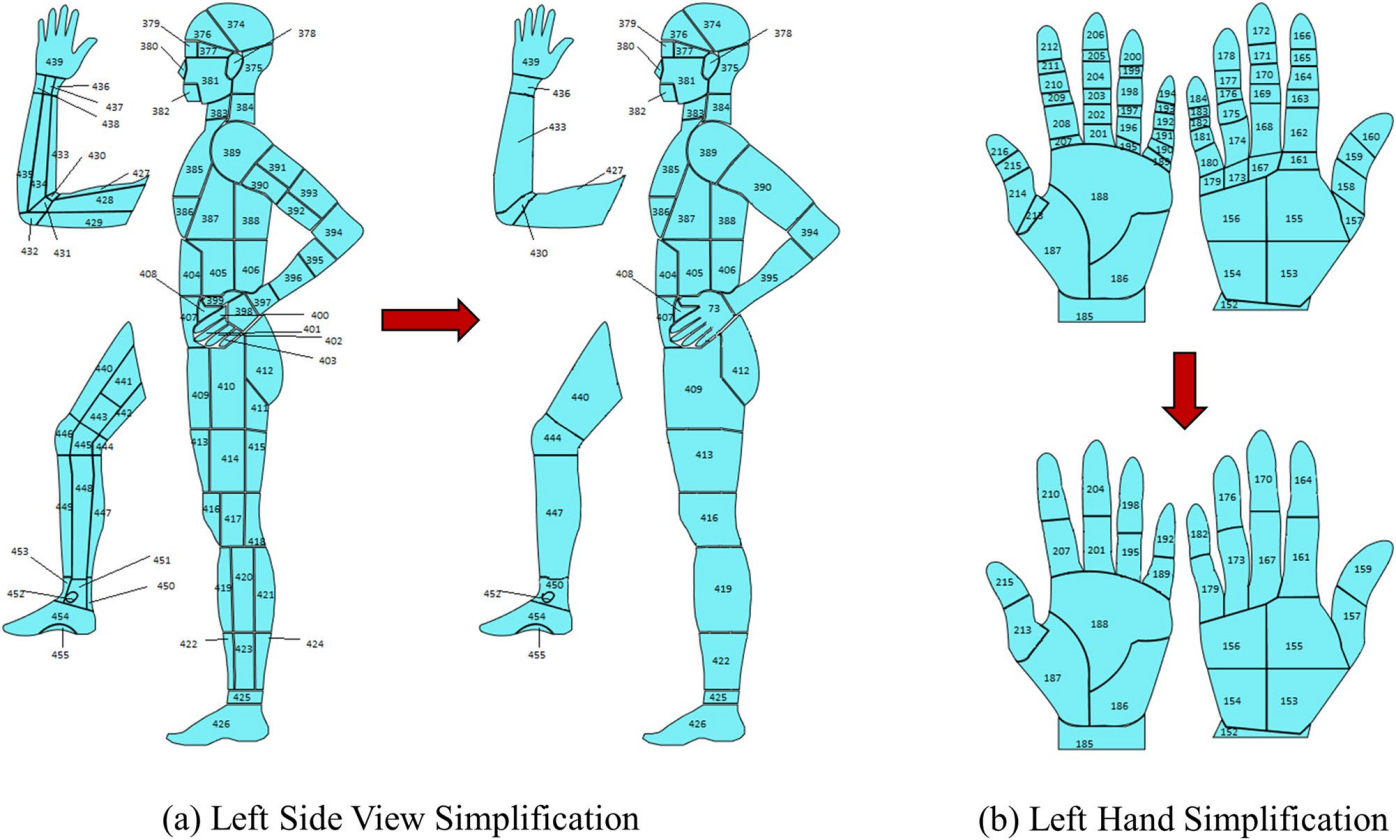
Body map simplification.

Through experiments, we observed that our number of data (images) is deficient regarding the different wound types and locations, leading to very few data points per class. To maintain the reliability of the experiment, the body map was further simplified by merging different sections of our developed body map. For example, body locations 436, 437, and 438 were combined and referenced as 436; similarly, body locations 390, 391, 392, and 393 were merged and referenced as 390, and so on. With this simplification, 161 location points were removed from our developed body map, and the total number of locations decreased from 484 to 323. This made our location classifier predict more realistic results, making the whole experiment reliable. More details are discussed in the “Selecting best experimental setup” section. Some examples of simplified body map are shown in Fig. 3. Our developed original body map is discussed here because, with the increment of the number of images, we will use this body map with 484 body locations in the future. For this research, we used the simplified body map containing 323 locations.

### Dataset processing

**Figure 4:**
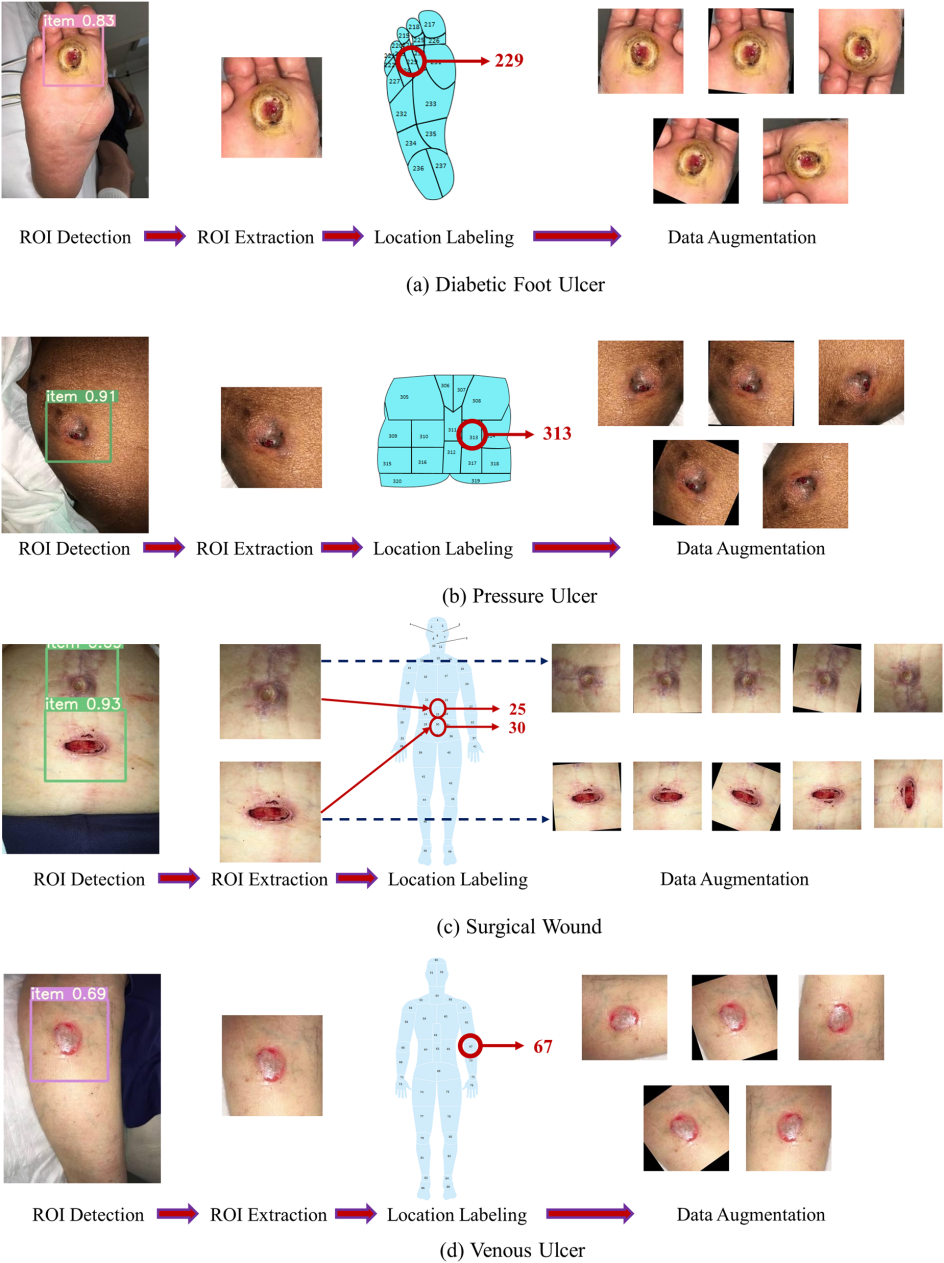
Dataset processing steps.

All datasets go through three significant steps: region of interest (ROI) cropping, location labeling, and data augmentation. The ROI of a wound image means the wound and some of its surrounding area (healthy skin) that contains the essential information of a wound. From each image, single or multiple ROIs were automatically cropped using our developed wound localizer^26^. The extracted ROIs are rectangular, but their height and weight differ depending on the wound size. Then all the ROI’s locations were labeled by a wound specialist at the AZH wound and vascular center. The location labeling was done by using our developed body map. As our body map represents each location with a unique number, each ROI was tagged with a location number for model training. Finally, rotation and flipping augmentations were used for each ROI to increase the data numbers. A total of five augmentations were applied to each ROI: horizontal and vertical flip, 25-degree, 45-degree, and 90-degree rotations. As wound location does not change with image augmentation, the location number was repeated for each augmented image. We also tried adding Gaussian noise and blurring augmentations which did not produce good ROIs, for which we discarded those augmentations. Figure 4 illustrates dataset processing steps.

From Fig. 4, we can see that the augmentation is done on the extracted ROIs. If we augmented the original images, the ROI cropping step would be more expensive. As our localization model is detecting bounding boxes, 25- and 45-degree rotated images may produce some overlap between ROIs in case of multiple wounds in a single image. Also, the black areas around the augmented images are evenly distributed in all classes (as we are using 25- and 45-degree rotations in all classes), which did not produce any class dependencies during classification. Finally, the black area produced by augmentation is entirely black (RGB code of 000), which is not present in wounds or human skins.

Each dataset (ROI) was divided into 60% training, 15% validation, and 25% test sets. First, the 25% test set was created from a random selection of the wound images to ensure no overlap between training and test sets. The validation set was also created randomly during the time of training. Next, the 75% training and validation datasets were augmented, while test images did not go through data augmentation. Two non-wound classes, named normal skin and background, were created by manually cropping corresponding ROIs from the original images. A wound specialist did the location tagging for healthy skin. As the background ROIs do not represent any location of our developed body map, each ROI is tagged with a location number ‘− 1’. Table 2 shows the number of images of all three datasets. All the six classes, diabetic, venous, arterial + venous, pressure, surgical, background, and normal skin, are represented with the following abbreviations D, V, A + V, P, S, BG, and N, respectively.

**Table 2:** Description of all datasets.

Dataset	AZH			Medetec			AZHMT		
Class	Training + validation	Test	Total	Training + validation	Test	Total	Training + validation	Test	Total
Background (BG)	450	25	475	0	0	0	450	25	475
Normal Skin (N)	450	25	475	0	0	0	450	25	475
Diabetic (D)	834	46	880	330	19	349	1164	65	1229
Pressure (P)	600	34	634	822	46	868	1422	80	1502
Surgical (S)	732	42	774	0	0	0	732	42	774
Venous (V)	1110	62	1172	0	0	0	0	0	0
Arterial + Venous (A + V)	0	0	0	456	25	481	1566	87	1653
Total	4176	234	4410	1608	90	1698	5784	324	6108

### Model

We see that our dataset contains both image and categorical (wound location) data from the above discussion. We used Keras Functional API^27^ to develop a network that can handle multiple inputs and mixed data. The Functional API is more flexible than the Sequential API, which can control models with non-linear topology, shared layers, and even multiple inputs or outputs. Considering a deep learning model as a directed acyclic graph (DAG) of layers, the functional API is a way to build graphs of layers.

**Figure 5:**
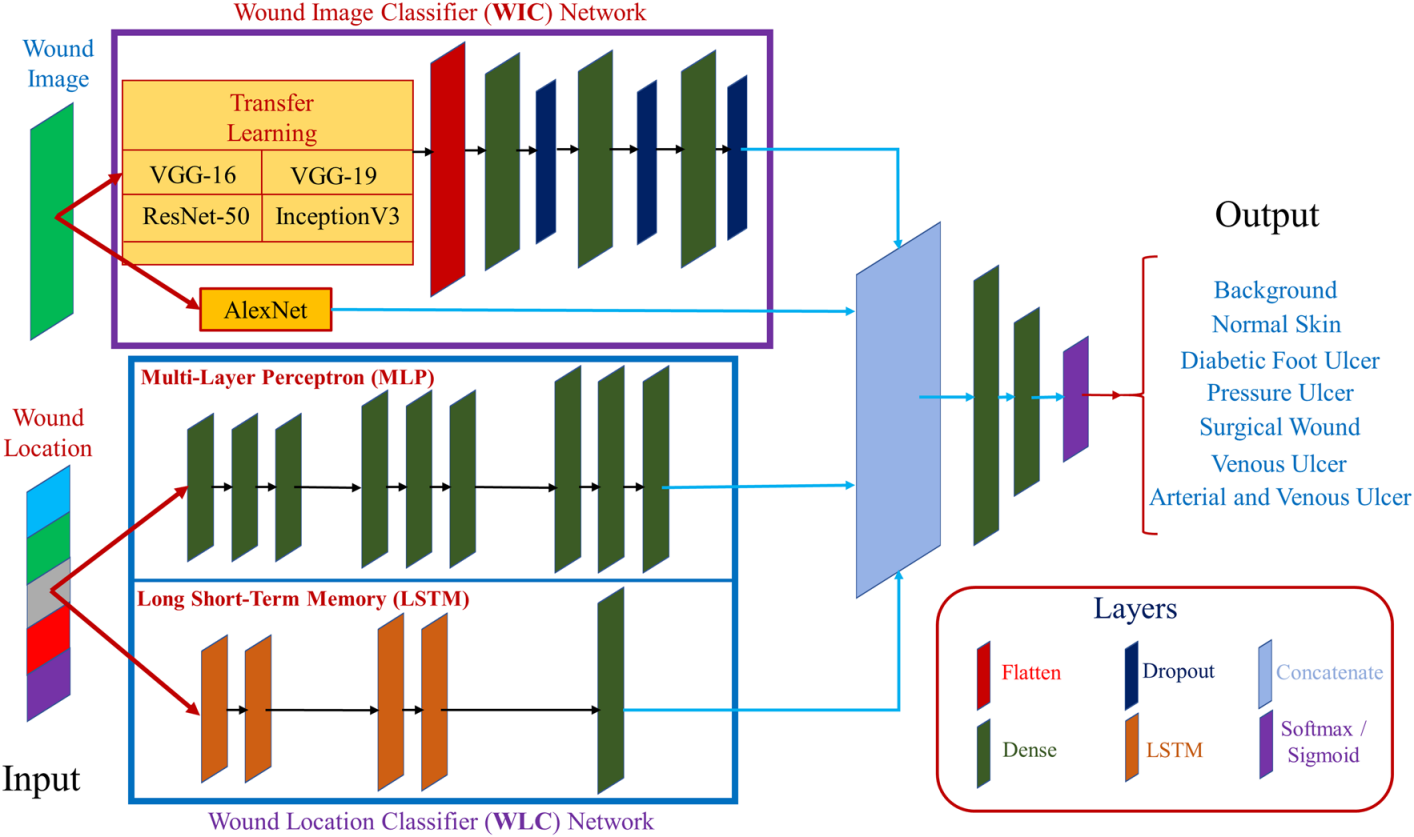
Wound multimodality classifier (WMC) network architecture.

Figure 5 shows the architecture of our wound-type classification network. Two separate neural networks for each data type were used to work with both image and location data. These networks were then considered input branches, and their outputs were combined into a final neural network. We address the image network as Wound Image Classifier (WIC) network, the location network as Wound Location Classifier (WLC) network, and the combined network as Wound Multimodality Classifier (WMC) network. The output of this WMC network is the probability of the wound class.

It is imperative for the multi-modal network (WMC) to arrange the data in the correct order. The output for the image and location data must be consistent, so the final combined (WMC) neural network must be fed with the right ordered data simultaneously. For example, to train the WMC network properly, we gave the output of the WIC network for the 148th DFU image and the output of the WLC network for the 148th DFU wound’s location as the input at the same time to the WMC network. If the data were not ordered correctly, the WMC network might have the WIC network’s output for the 148th DFU image and the WLC network’s output for the 55th PU wound’s location as input at the same time, which will lead to a wrong classification. This arrangement was taken care of by giving each ROI a unique index number and tagging the corresponding location to that index number.

#### Wound image classifier (WIC) network

The wound image classifier (WIC) network was built upon transfer learning, except the AlexNet^28^. Transfer learning means taking advantage of features learned on one problem and using them in another similar situation. This method is proper when the dataset in hand is small in number to train a full-scale model from scratch, and the memory power is limited to train a vast deep learning model. The most commonly used workflow of transfer learning is: (1) take a previously trained model’s layers, (2) freeze the layers, (3) add some new, trainable layers on top of the frozen layers, which will learn to turn the old features into predictions on a new dataset, and (4) train the new layers on the new dataset^29^. There are 26 deep learning models in Keras Applications^30^, among which we chose four top-rated classification models: VGG16^31^, VGG19^32^, ResNet50^33^, and InceptionV3^34^; and took their previously trained layers to apply transfer learning. All the layers, except the top layer, were frozen for all these four models, and three Dense layers with dropout layers were added (Fig. 5, top WIC box) for training on our wound datasets. All three Dense layers contain 512 trainable neurons, with all having the ReLU activation. The AlexNet^28^ was implemented following the original architecture. The output layer was added with either softmax or sigmoid layer for multi-class or binary-class classification for all the models, respectively.

#### Wound location classifier (WLC) network

The wound location classifier (WLC) network can classify wound locations using either a Multi-Layer Perceptron (MLP) or Long Short-Term Memory (LSTM) network. As the location data is categorical, we used one-hot encoding to represent the data, representing each input to the WLC network as a one-hot vector. The WLC network handles only one categorical data (location), for which the architecture of the network was kept simple. With a deeper network, the accuracy did not improve (sometimes decreases), and resources (time and memory) became expensive. The MLP network contains nine Dense layers, all having the ReLU activation. The first three layers contain 128 neurons, the following three layers contain 256 neurons, and the last three layers contain 512 neurons (Fig. 5, middle MLP box). The LSTM contains four LSTM layers, followed by a Dense layer, with all having the ReLU activation. The first two layers contain 32 neurons, followed by two LSTM layers having 64 neurons each, and finally, the Dense layer contains 512 neurons (Fig. 5, bottom LSTM box). The output layer was added with either softmax or sigmoid layer for multi-class or binary-class classification for all the models, respectively.

#### Wound multimodality classifier (WMC) network

As discussed earlier, the Wound Multimodality Classifier (WMC) network was designed using Keras Functional API^27^, which can predict the wound classes based on both wound image and location information. At first, the image data went through the WIC network, the location data went through the WLC network, and the outputs of the networks were concatenated. Then, two Dense layers were added after concatenation to learn from the merged features. These Dense layers contain 512 and 256 neurons, respectively. Finally, the output layer was added with either a softmax or sigmoid layer for multi-class or binary-class classification.

## Experiment and result and discussion

### Experimental setup

Lots of experiments were performed with different setups. Classification between D vs. V, D vs. S, N vs. D, etc. are some examples of binary classification, and D vs. P vs. S, BG vs. N vs. S vs. V, BG vs. N vs. D vs. P vs. S vs. V, etc. are some examples of multi-class classification. In the WMC network, all combinations of the WIC and WLC networks (AlexNet + MLP, AlexNet + LSTM, ResNet50 + MLP, VGG16 + LSTM, etc.) were applied for the four wound class classification (D vs. P vs. S vs. V) on the AZH dataset. Based on the results (discussed later), the best two combinations were applied for the other multi-modal classifications.

All the models were written in Python programming language using the Keras deep learning framework and trained on an Nvidia GeForce RTX 2080Ti GPU platform. All models were trained for 250 epochs with a batch size of 25, a learning rate of 0.001, and an Adam optimizer. Two callbacks were used with the best validation accuracy and the best combination of validation and training accuracy saving. For multi-class and binary class classification, *sparse_categorical_crossentropy* and *binary_crossentropy* loss functions are used, respectively.

To investigate the classification performance, we used accuracy as the performance metric. Accuracy is the ratio of correctly predicted data to the total amount of data. To evaluate binary classifications, we used precision, recall, and f1-score as performance metrics as well. Equations (1) to (4) show the related formulae for these evaluation metrics. In these equations, TP, TN, FP, and FN, represent True Positive, True Negative, False Positive, and False Negative measures. More details about these equations can be found in^35^.

1 $$Accuracy= \frac{TP+TN}{TP+FP+FN+TN}$$

2 $$Precision= \frac{TP}{TP+FP}$$

3 $$Recall= \frac{TP}{TP+FN}$$

4 $$F1{-}Score= 2\times \frac{Recall\times Precision}{Recall+Precision}$$

### Results

#### Selecting best experimental setup

Four wound class classification (D vs. P vs. S vs. V) on the AZH dataset was chosen to select the best combinations for the WMC network. This classification was the most challenging classification task, as there were no normal skin (N) or background (BG) images in the experiment. This experiment was done with our originally developed body map, which contains 484 locations. Table 3 shows the results of this experiment. We also present the results on the original dataset (without any augmentation) for this experiment to show the effect (improvement) of data augmentation. The performances of MLP and LSTM were similar on the WLC network, and the VGG16 and VGG19 performed best on the WIC network. Their combinations: VGG16 + MLP, VGG19 + MLP, VGG16 + LSTM, and VGG19 + LSTM, also worked best for the WMC network. The performance of AlexNet + MLP, AlexNet + LSTM, ResNet50 + MLP, and ResNet50 + LSTM were very poor. The InceptionV3 + MLP and InceptionV3 + LSTM performances were also not good enough to apply to all the experiments. Running all these combinations for many experiments was also expensive (both with time and memory). So, from these results, we applied the best four combinations (VGG16 + MLP, VGG19 + MLP, VGG16 + LSTM, and VGG19 + LSTM) for all the remaining experimental setups.

**Table 3:** Four wound class classification (D vs. P vs. S vs. V) on AZH dataset with original body map.

Input	Model	Original dataset	Augmented dataset
		Accuracy (%)	Accuracy (%)
Location	MLP	66.30	71.74
	LSTM	66.85	72.28
Image	AlexNet	35.33	37.50
	VGG16	65.76	71.73
	VGG19	56.52	63.04
	InceptionV3	51.09	56.52
	ResNet50	33.70	33.70
Image + location	AlexNet + MLP	55.43	61.41
	VGG16 + MLP	77.17	78.
	VGG19 + MLP	62.50	72.28
	InceptionV3 + MLP	61.41	70.11
	ResNet50 + MLP	63.04	66.85
	AlexNet + LSTM	58.15	66.85
	VGG16 + LSTM	72.83	**79.35**
	VGG19 + LSTM	71.20	76.63
	InceptionV3 + LSTM	64.67	69.02
	ResNet50 + LSTM	33.70	34.79

The bold represents the highest results/accuracy achieved for each experiment.

The same four wound-class classification (D vs. P vs. S vs. V) on the AZH dataset was done with the simplified body map, which contains 323 locations. Table 4 shows the comparison of this experiment’s result with the previous result (shown in Table 3). The image classifier (WIC) has no effect on the change in the body map, for which it was excluded from Table 4. With improved accuracy in all models, we used the simplified body map for all the remaining experiments.

**Table 4:** Four wound class classification (D vs. P vs. S vs. V) on AZH dataset with simplified body map.

Input	Model	Accuracy with original body map (%)	Accuracy with simplified body map (%)
Location	MLP	71.74	74.46
	LSTM	72.28	73.37
Image + location	VGG16 + OHV	N/A	77.72
	VGG19 + OHV	N/A	73.91
	VGG16 + MLP	78.26	**81.52**
	VGG19 + MLP	72.28	78.80
	VGG16 + LSTM	**79.35**	80.43
	VGG19 + LSTM	76.63	79.89

The bold represents the highest results/accuracy achieved for each experiment.

We also tried giving the one hot vector (OHV) directly to the dense layer of the CNN, but it produced poor results than passing it through the MLP or LSTM (VGG16 + OHV and VGG19 + OHV in Table 4). Also, we want to see the comparison between image-based, location-based, and multimodality classifications; if we use the one hot vector directly, then we do not have any location classifier (WIC) to make the comparison. For this reason, OHV was not directly combined with CNN layers for the rest of the experiments.

#### Experiment on AZH dataset

A classification between all the classes was performed on the AZH dataset. Table 5 shows the results of this six-class classification (BG vs. N vs. D vs. P vs. S vs. V). We achieved the highest accuracy of 82.48% with the multi-modal (WMC) network using the VGG19 + MLP combination, where the highest accuracies reached from WLC and WIC networks are 67.52% and 75.64% using LSTM and VGG16 networks, respectively.

**Table 5:** Six-class classification (BG vs. N vs. D vs. P vs. S vs. V) on AZH dataset.

Input	Model	Accuracy (%)
Location	MLP	64.96
	LSTM	67.52
Image	VGG16	75.64
	VGG19	64.96
Image + location	VGG16 + MLP	79.49
	VGG19 + MLP	**82.48**
	VGG16 + LSTM	79.91
	VGG19 + LSTM	72.22

The bold represents the highest results/accuracy achieved for each experiment.

Four five-class classifications were performed on the AZH dataset. The classifications were (1) BG vs. N vs. D vs. P vs. V, (2) BG vs. N vs. D vs. S vs. V, (3) BG vs. N vs. D vs. P vs. S, and (4) BG vs. N vs. P vs. S vs. V. We achieved the highest accuracy of 86.46%, 91.00%, 83.14%, and 86.17% for classification number (1), (2), (3), and (4), respectively. In all four classifications, the highest accuracy was achieved with the multi-modal (WMC) networks. Table 6 shows the detailed results of these classifications.

**Table 6:** Four five-class classifications on AZH dataset.

Classifications		BG–N–D–P–V	BG–N–D–S–V	BG–N–D–P–S	BG–N–P–S–V
Input	Model	Accuracy (%)	Accuracy (%)	Accuracy (%)	Accuracy (%)
Location	MLP	67.71	75.00	59.30	69.68
	LSTM	68.75	72.00	59.30	71.81
Image	VGG16	69.79	70.50	64.53	75.53
	VGG19	76.56	74.50	67.44	72.34
Image + location	VGG16 + MLP	**86.46**	85.00	**83.14**	84.04
	VGG19 + MLP	85.42	86.50	77.33	**86.17**
	VGG16 + LSTM	84.38	**91.00**	77.33	77.13
	VGG19 + LSTM	78.65	89.50	73.26	75.00

The bold represents the highest results/accuracy achieved for each experiment.

Six four-class classifications were performed on the AZH dataset, along with one wound class classification (shown in Tables 3 and 4). The classifications were: (1) BG vs. N vs. D vs. V, (2) BG vs. N vs. P vs. V, (3) BG vs. N vs. S vs. V, (4) BG vs. N vs. D vs. P, (5) BG vs. N vs. D vs. S, and (6) BG vs. N vs. P vs. S. We achieved the highest accuracy of 95.57%, 92.47%, 94.16%, 89.23%, 91.30%, and 85.71% for classification number (1), (2), (3), (4), (5), and (6), respectively. In all six classifications, the highest accuracy was achieved with the multi-modal (WMC) networks. Table 7 shows the detailed results of these classifications.

**Table 7:** Six four-class classifications on AZH dataset.

Classifications		BG–N–D–V	BG–N–P–V	BG–N–S–V	BG–N–D–P	BG–N–D–S	BG–N–P–S
Input	Model	Accuracy (%)					
Location	MLP	76.58	73.29	77.27	65.38	71.74	69.04
	LSTM	78.48	76.03	83.12	64.62	73.91	67.46
Image	VGG16	93.67	89.73	87.66	82.31	77.54	83.33
	VGG19	89.87	86.99	88.31	80.00	81.88	83.33
Image + location	VGG16 + MLP	94.30	91.78	**94.16**	86.15	86.96	**85.71**
	VGG19 + MLP	**95.57**	91.78	92.86	86.92	**91.30**	81.75
	VGG16 + LSTM	89.87	**92.47**	90.91	86.15	84.78	83.33
	VGG19 + LSTM	94.30	89.04	88.89	**89.23**	85.51	83.33

The bold represents the highest results/accuracy achieved for each experiment.

Four three-wound-class classifications were performed on the AZH dataset. The classifications were (1) D vs. S vs. V, (2) P vs. S vs. V, (3) D vs. P vs. S, and (4) D vs. P vs. V. We achieved the highest accuracy of 92.00%, 85.51%, 72.95%, and 84.51% for classification number (1), (2), (3), and (4), respectively. In all four wound-class classifications, the highest accuracy was achieved with the multi-modal (WMC) networks. Table 8 shows the detailed results of these classifications.

**Table 8:** Four three-wound-class classifications on AZH dataset.

Classifications		D–S–V	P–S–V	D–P–S	D–P–V
Input	Model	Accuracy	Accuracy	Accuracy	Accuracy
Location	MLP	81.33	82.61	65.57	78.87
	LSTM	82.00	80.43	68.85	78.87
Image	VGG16	74.67	68.12	61.48	76.06
	VGG19	76.00	70.23	58.20	68.31
Image + location	VGG16 + MLP	85.33	**85.51**	70.49	80.28
	VGG19 + MLP	**92.00**	82.61	71.31	**84.51**
	VGG16 + LSTM	80.67	81.88	**72.95**	83.10
	VGG19 + LSTM	87.33	68.12	67.21	**84.51**

The bold represents the highest results/accuracy achieved for each experiment.

Ten binary classifications were performed on the AZH dataset. The classifications were: (1) N vs. D, (2) N vs. P, (3) N vs. S, (4) N vs. V, (5) D vs. P, (6) D vs. S, (7) D vs. V, (8) P vs. S, (9) P vs. V, and (10) S vs. V. We achieved highest accuracy of 100%, 98.31%, 98.51%, 100%, 85.00%, 89.77%, 94.44%, 89.47%, 90.63%, and 97.12% for classification number (1), (2), (3), (4), (5), (6), (7), (8), (9), and (10), respectively. In all binary classifications, the highest accuracy was achieved with the multi-modal (WMC) networks. Table 9 shows the detailed results of these binary classifications. The precision, recall, and f1-score for all the best models (according to accuracy) are also calculated and shown in Table 10.

**Table 9:** Accuracy of ten binary classifications on AZH dataset.

Classifications		N–D	N–P	N–S	N–V	D–P	D–S	D–V	P–S	P–V	S–V
Input	Model	Accuracy									
Location	MLP	78.87	64.41	74.63	78.16	78.75	87.50	89.81	73.68	87.50	93.27
	LSTM	77.46	43.37	76.12	78.16	78.75	81.82	57.41	73.68	85.42	93.27
Image	VGG16	98.59	96.61	97.01	98.85	81.25	79.55	87.96	77.63	84.38	84.62
	VGG19	98.59	**98.31**	97.01	98.85	71.25	80.68	87.96	73.68	86.46	86.54
Image + location	VGG16 + MLP	97.18	96.61	**98.51**	98.85	80.00	**89.77**	**94.44**	**89.47**	88.54	94.23
	VGG19 + MLP	95.77	94.92	97.01	98.85	80.00	84.10	92.59	80.26	**90.63**	**97.12**
	VGG16 + LSTM	97.18	96	95.52	98.85	83.75	80.68	**94.44**	76.32	83.33	84.62
	VGG19 + LSTM	**100**	**98.31**	97.01	**100**	**85.00**	77.27	88.89	71.05	82.29	79.81

The bold represents the highest results/accuracy achieved for each experiment.

**Table 10:** Precision, recall, and F1-scores of the best models of ten binary classifications on AZH dataset.

Classifications	Best model(s)	Precision (%)	Recall (%)	F1-score (%)
N–D	VGG19 + LSTM	100	100	100
N–P	VGG19 + LSTM	100	97.06	98.51
N–S	VGG16 + MLP	100	97.62	98.80
N–V	VGG19 + LSTM	100	100	100
D–P	VGG19 + LSTM	76.19	94.12	84.21
D–S	VGG16 + MLP	83.67	97.62	90.11
D–V	VGG16 + MLP	92.42	98.39	95.31
	VGG16 + LSTM	92.42	98.39	95.31
P–S	VGG16 + MLP	86.96	95.24	90.91
P–V	VGG19 + MLP	88.41	98.39	93.13
S–V	VGG19 + MLP	95.38	100	97.64

#### Experiment on Medetec dataset

A classification between all the classes was performed on the Medetec dataset. Table 11 shows the results of this three-wound-class classification (D vs. P vs. A + V). We achieved the highest accuracy of 86.67% with the multi-modal (WMC) network using the VGG19 + MLP and VGG19 + LSTM combinations, where the highest accuracy achieved from WLC and WIC networks was 85.56% and 82.22% using both MLP and LSTM, and VGG16 networks, respectively.

**Table 11:** Three-wound-class classification (D vs. P vs. A + V) on Medetec dataset.

Input	Model	Accuracy (%)
Location	MLP	85.56
	LSTM	85.56
Image	VGG16	82.22
	VGG19	77.78
Image + location	VGG16 + MLP	85.56
	VGG19 + MLP	**86.67**
	VGG16 + LSTM	85.56
	VGG19 + LSTM	**86.67**

The bold represents the highest results/accuracy achieved for each experiment.

#### Experiment on AZHMT dataset

A classification between all the classes was performed on the AZHMT dataset. Table 12 shows the results of this six-class classification (BG vs. N vs. D vs. P vs. S vs. A + V). We achieved the highest accuracy of 83.04% with the multi-modal (WMC) network using the VGG19 + LSTM combination. The highest accuracy achieved from WLC and WIC networks was 71.30% and 72.22% using LSTM and VGG19 networks, respectively.

**Table 12:** Six-class classification (BG vs. N vs. D vs. P vs. S vs. A + V) on AZHMT dataset.

Input	Model	Accuracy (%)
Location	MLP	69.44
	LSTM	71.30
Image	VGG16	67.59
	VGG19	72.22
Image + location	VGG16 + MLP	81.17
	VGG19 + MLP	81.79
	VGG16 + LSTM	72.22
	VGG19 + LSTM	**83.04**

The bold represents the highest results/accuracy achieved for each experiment.

A four-wound-class classification was performed on the AZHMT dataset. The classification was done among the D, P, S, and A + V classes. We achieved the highest accuracy of 84.31% with the multi-modal (WMC) network using the VGG19 + MLP combination. The highest accuracy achieved from WLC and WIC networks was 78.83% and 68.61% using LSTM and VGG16 networks, respectively. Table 13 shows the detailed results of this four-wound-class classification.

**Table 13:** Four-wound-class classification (D vs. P vs. S vs. A + V) on AZHMT dataset.

Input	Model	Accuracy (%)
Location	MLP	78.10
	LSTM	78.83
Image	VGG16	68.61
	VGG19	63.14
Image + location	VGG16 + MLP	79.56
	VGG19 + MLP	**84.31**
	VGG16 + LSTM	68.25
	VGG19 + LSTM	68.98

The bold represents the highest results/accuracy achieved for each experiment.

#### Cross-validation on AZH dataset

Several cross-validation (CV) experiments were performed on the AZH dataset to prove the reliability of this study. fivefold cross-validations were performed using sklearn’s StratifiedKFold method with shuffle set to ‘True’. The most challenging tasks from all classifications performed on the AZH dataset were chosen for this CV experiment. For example, one of the selected experiments was the D vs. P ulcer classification, which had the lowest accuracy among all binary classifications (Table 9). Also, WMC models with the best performance and their corresponding WIC and WLC models were chosen only due to time and memory limitations. Finally, we performed external validation on the Medetec dataset. From Table 2, the only common classes between AZH and Medetec datasets are D and P; and as we do not have any other public wound dataset available, only this experiment (D vs. P) was chosen for external validation. For result comparison, we also performed this external validation on the best model we generated using the holdout test set experiment. Table 14 shows the detailed results of all cross-validation experiments.

**Table 14:** Cross-validation on the AZH dataset.

Experiments	Model	Accuracy						
		Holdout test set	Cross-validation test					
			Fold1	Fold2	Fold3	Fold4	Fold5	Average
BG vs. N vs. D vs. P vs. S vs. V	MLP	64.96	70.43	63.99	64.52	70.97	74.19	68.82
	VGG19	64.96	59.68	61.29	53.76	63.98	62.90	60.32
	VGG19 + MLP	**82.48**	80.62	74.73	**80.65**	78.49	75.27	**77.95**
BG vs. N vs. D vs. P vs. S	MLP	59.30	77.93	54.74	64.96	64.71	63.97	65.26
	VGG16	64.53	74.45	70.07	62.04	58.82	74.26	67.93
	VGG16 + MLP	**83.14**	**83.21**	73.22	79.56	75.00	79.41	**78.08**
D vs. P vs. S vs. V	MLP	74.46	65.07	72.60	69.18	77.40	67.12	70.27
	VGG16	71.73	63.01	60.96	60.96	69.18	58.22	62.47
	VGG16 + MLP	**81.52**	71.23	73.97	76.03	**82.88**	67.81	**74.38**
D vs. P vs. S	LSTM	68.85	62.88	67.01	62.89	73.96	76.04	68.56
	VGG16	61.48	63.92	71.13	70.10	64.58	62.50	66.45
	VGG16 + LSTM	**72.95**	70.10	74.23	72.16	75.00	**78.13**	**73.92**
D vs. P	LSTM	78.75	75.00	68.75	78.13	78.13	76.19	75.24
	VGG19	71.25	71.88	65.63	79.69	70.31	66.67	70.84
	VGG19 + LSTM	**85.00**	78.13	70.31	**81.25**	79.69	79.37	**77.75**
External validation	VGG19 + LSTM	**74.71**	59.14	59.53	57.20	**83.27**	79.77	**67.78**

The bold represents the highest results/accuracy achieved for each experiment.

### Result comparison with previous works

Classification results depend on many factors like dataset, model, training-validation-testing split, balanced or unbalanced dataset, resources used for training, etc. Though the datasets and other factors between our work and previous classification works are not the same, this section mainly focuses on how the multimodality using both image and location data can improve the classification accuracy. The comparison with the previous works was only made if all the classes of that work’s dataset were present in our dataset. Our previous work^16^’s dataset is most similar to the work presented in this manuscript. Alongside^16^, the classifications performed in^12,13^, and^15^ have the classes that are present in our dataset. A detailed comparison between previous works and our current work is shown in Table 15.

**Table 15:** Comparison among the previous works and the present work.

Work	Classification	Evaluation metrics	Previous work			Present work		
			Model	Dataset	Result (%)	Model	Dataset	Result (%)
Goyal et al.^12^	Healthy skin vs. DFU skin (N vs. D)	Accuracy	DFUNet	A dataset containing 397 wound images	92.5	VGG19 + LSTM	AZH	**100**
Aguirre et al.^13^	VLU versus non-VLU (N vs. V, D vs. V, P vs. V, S vs. V)	Accuracy	VGG19	A dataset of 300 wound images	85	**N-V:** VGG19 + LSTM	AZH	**100**
						**D-V:** VGG16 + MLP & VGG16 + LSTM		**94.44**
						**P–V:** VGG19 + MLP		**90.63**
						**S-V:** VGG19 + MLP		**97.12**
Alzubaidi et al.^15^	Normal skin vs. abnormal (DFU) skin (N vs. D)	F1-Score	DFU_QUTNet + SVM	A dataset containing 754-foot images	94.5	VGG19 + LSTM	AZH	**100**
Rostami et al.^16^	S–V	Accuracy	An end-to-end Ensemble DCNN-based Classifier	A new dataset containing 538 wound images	96.4	VGG19 + MLP	AZH	**97.12**
	D–S–V				91.9	VGG19 + MLP		**92.00**
	BG–N–D–V				89.41	VGG19 + MLP		**95.57**
	BG–N–P–V				86.57	VGG16 + LSTM		**92.47**
	BG–N–S–V				92.20	VGG16 + MLP		**94.16**
	BG–N–D–P				80.29	VGG19 + LSTM		**89.23**
	BG–N–D–S				90.98	VGG19 + MLP		**91.30**
	BG–N–P–S				84.12	VGG16 + MLP		**85.71**
	BG–N–D–P–V				79.76	VGG16 + MLP		**84.46**
	BG–N–D–S–V				84.94	VGG16 + LSTM		**91.00**
	BG–N–D–P–S				81.49	VGG16 + MLP		**83.14**
	BG–N–P–S–V				83.53	VGG19 + MLP		**86.17**
	BG–N–D–P–S–V				68.69	VGG19 + MLP		**82.48**

The bold represents the highest results/accuracy achieved for each experiment.

The reasons why other related works were not considered in this comparison are^10^: performs burn vs. pressure ulcer classification, and our datasets do not contain any burn images^11^; performs binary classification of ischemia vs. non-ischemia and infection vs. non-infection on DFU images, which is not compatible with our datasets^14^; performs binary classifications between such kind of wounds (wound, infection (SSI), granulation tissue, etc.), which are not present in our datasets; and^17^ performs multi-class wound classifications among diabetic, lymphovascular, pressure injury, and surgical wounds and our datasets do not contain the lymphovascular wound type.

## Discussion

In all the experiments performed in this manuscript, there were two types of classifications: (1) mixed-class classifications (e.g., three-class classification, five-class classification, etc.), and (2) wound-class classifications (e.g., four wound-class classifications, three wound-class classifications, etc.). The wound-class classification did not contain any non-wound classes (i.e., normal skin and background), and they were more challenging to classify than the mixed-class classification. This section will discuss the classification’s performances, comparison with state-of-the-art results, limitations, and how to overcome them.

### Performance analysis and the power of multimodality

On the AZH dataset, for mixed-class classifications, we performed one six-class, four five-class, six four-class, and four binary classifications; and for wound-class classifications, we performed one four-wound-class, four three-wound-class, and six binary classifications. From Tables 5, 6, 7, 8, and 9, the same consistency of the model performances is observed, where the best to worst results were achieved by WMC, WIC, and WLC classifiers, respectively. Though a single model of WLC or WIC or a single combination of WMC did not always produce the best performance, the WMC classifier always performed the best in comparison to the WIC or WLC classifiers. The same pattern can also be seen in the wound class classifications. Also, in most cases, when using only location data, we got lower accuracy for the wound classification (Tables 5, 6, 7, 9) compared to using only image data, which indicates that the data is not location-dependent.

**Figure 6:**
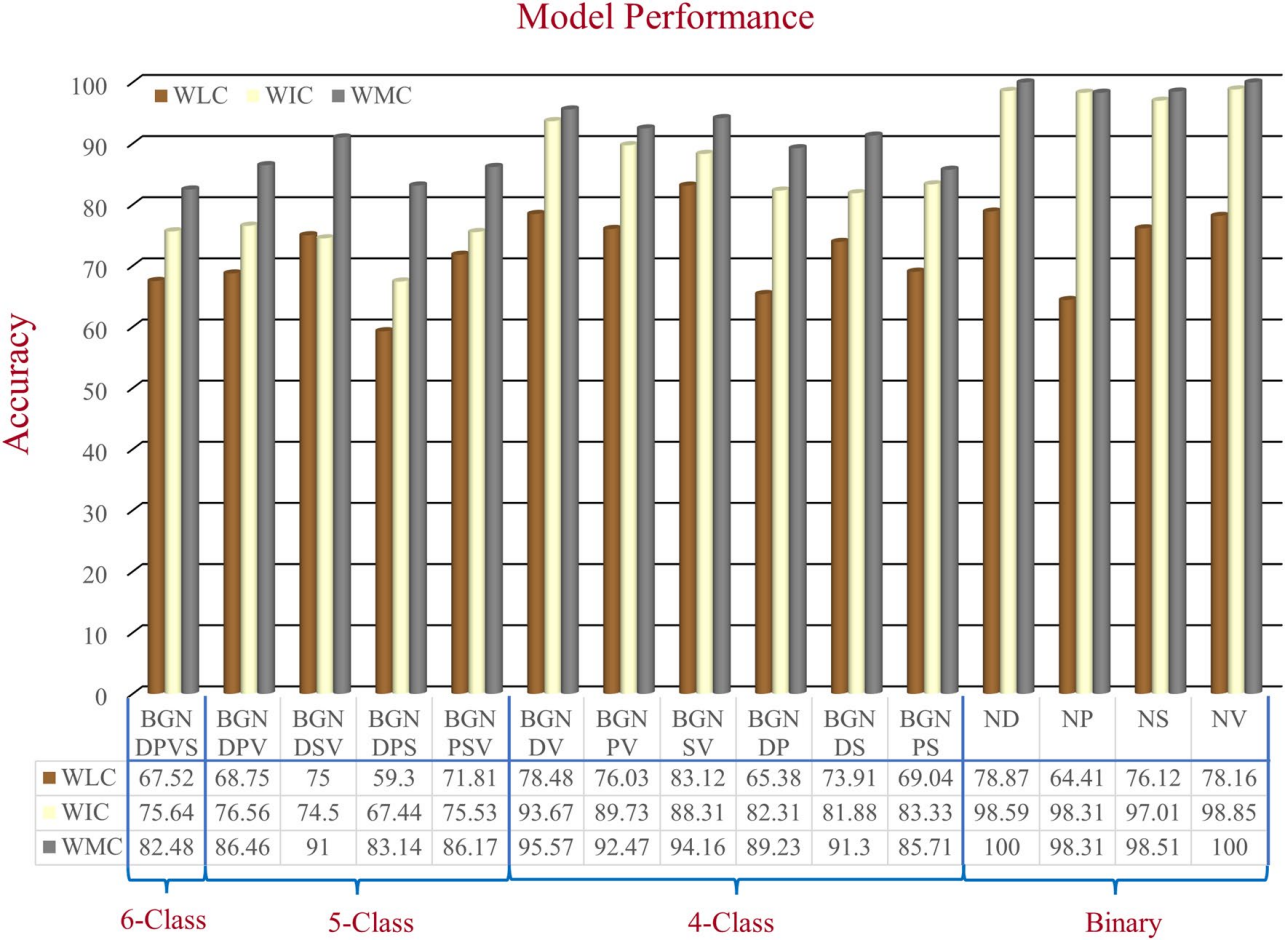
Performance comparison of mixed-class classification among the best models from each category (location—WLC, image—WIC, and multimodality—WMC) on AZH dataset.

**Figure 7:**
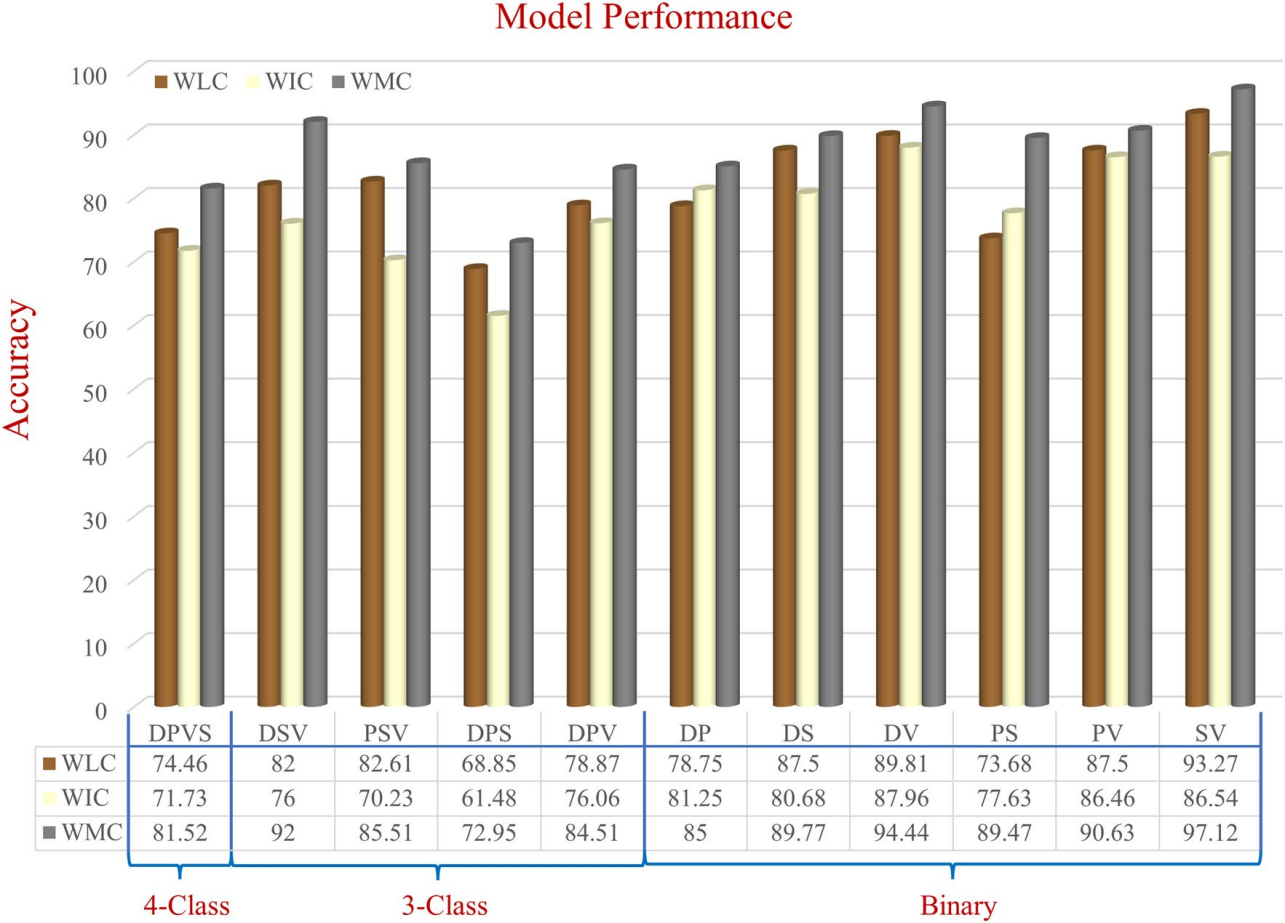
Performance comparison of wound-class classification among the best models from each category (location—WLC, image—WIC, and multimodality—WMC) on AZH dataset.

The performance comparison of mixed-class classifications among the best models from each category (location, image, and multimodality) is shown in Fig. 6. The performance comparison among the best models of wound-class classifications from each category (location, image, and multimodality) is shown in Fig. 7. From Fig. 6, the lowest accuracy was produced by BGNDPS (83.14%), and from Fig. 7, the most insufficient accuracy was produced by DPS (72.95%). So, separating diabetic, pressure, and the surgical wound was the hardest, according to our experiments. Also, from Fig. 7, among all binary classifications, D vs. P had the lowest accuracy of 85%. So, we can say that differentiation between diabetic and pressure wounds was the most complicated task. From Fig. 6, the highest accuracy was achieved by ND, NP, NS, and NV classifications with 100%, 98.31%, 98.51%, and 100%, respectively. Also, from Fig. 7, the highest accuracy was achieved by SV classification with 97.12% accuracy. So, differentiating between normal skin and other wound types (D, V, S, and P) and differentiating between surgical wounds and venous leg ulcers were the most straightforward classifications task for our developed WMC classifier. Finally, from Figs. 6 and 7, we can see that multimodality using wound image and location (WMC) performed best in comparison with single (image or location) modality (WLC or WIC) in all scenarios on the AZH dataset; and mixed-class classification results are comparatively higher than wound-class classification results.

### Robustness testing

To evaluate the robustness of our developed WMC classifier, we performed an experiment on a publicly available dataset named Medetec Dataset, which has a completely different data collection and distribution than our collected and developed AZH Dataset. On this dataset, we performed only one wound-type classification among all three classes (D, P, and A + V). The highest accuracies achieved by WLC, WIC, and WMC classifiers were 85.56%, 82.22%, and 86.67%, respectively. So, clearly, the highest accuracy was achieved by the WMC classifier, which proves that the WMC works well on different datasets with separate distributions.

### The effect of bigger dataset

**Figure 8:**
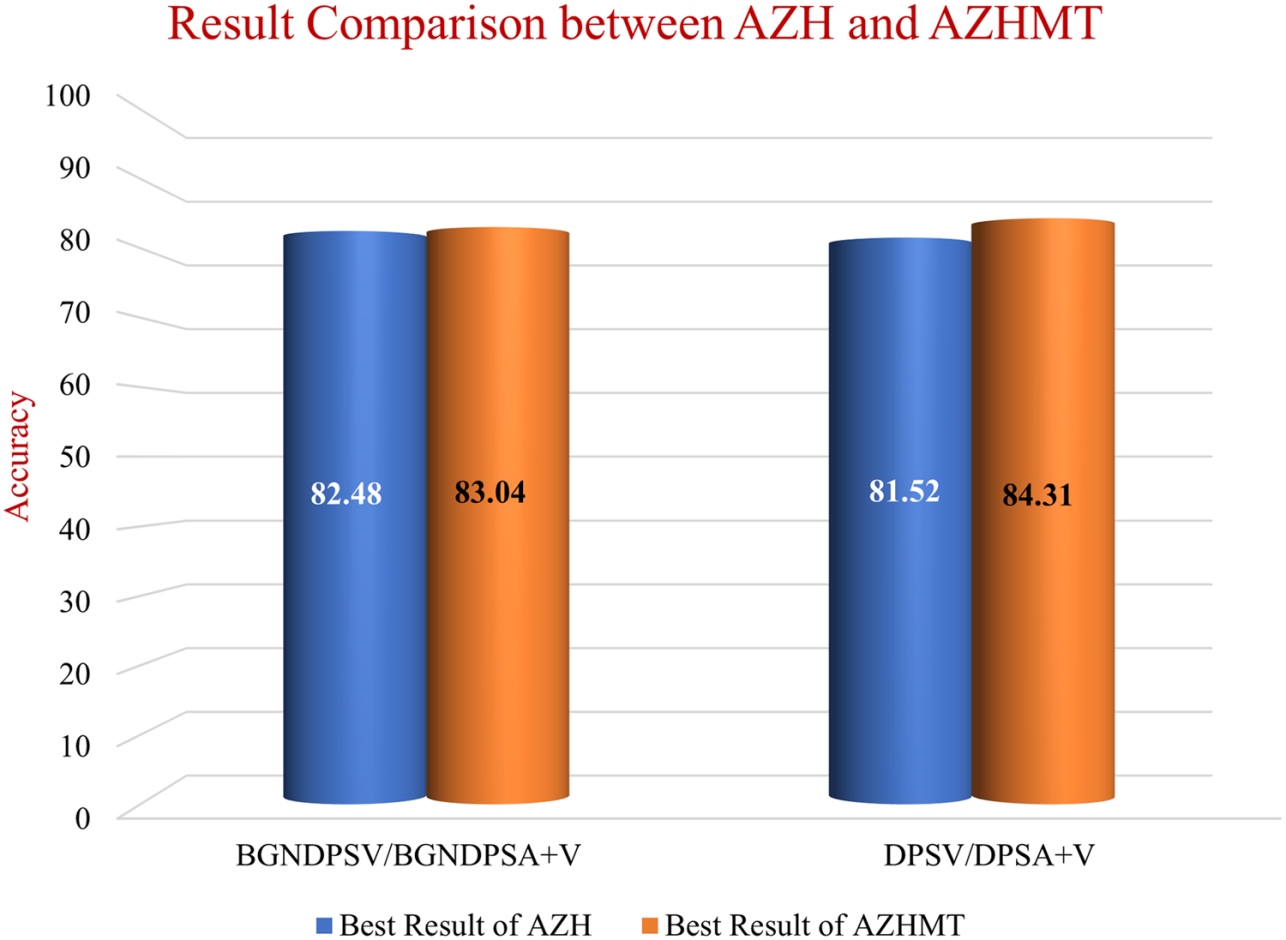
Comparison between the highest results (accuracy) of AZH and AZHMT datasets.

We developed a mixed and bigger dataset named AZHMT to test the effect of adding more data points to our model performance. AZHMT is a mixed dataset containing wound image and location data from AZH and Medetec datasets. On the AZHMT dataset, we performed one six-mixed-class classification (BG–N–D–P–S–A + V) and one four-wound-class classification (D–P–S–A + V). Comparing these results of AZH and AZHMT datasets, we see that with the AZHMT dataset, we achieved higher accuracy than the AZH dataset. A comparison between the highest results (accuracy) of AZH and AZHMT datasets is shown in Fig. 8. Both the results are from the multi-modal network (WMC), as it outperformed all the single modal (WIC and WLC) networks. For the six-class classification, the AZHMT dataset has 0.56% more accuracy than the AZH dataset. For the four-wound-class classification, the AZHMT dataset has 2.79% more accuracy than the AZH dataset. Here, AZHMT contains more data than the AZH dataset, which is an advantage for training deep learning models; but AZHMT also contains mixed data from two sources, which makes the dataset more challenging to classify; AZHMT also contains mixed data on a single class (arterial and venous ulcer combination), which may also impact the results. Regardless of some disadvantages of the mixed dataset, this comparison proves that increasing data points improve the model performance.

### Cross-validation results analysis

From Table 14, we achieved better results for specific folds compared to the holdout test data in 5, 4, and 3 class classifications. For 6 class and binary classifications, we got poor results in all fold performances. In average accuracy among all folds, except for 3 class classification, we had less accuracy for all other classifications. For specific folds, the accuracy got down by 1.83% and 3.75% for 6 class (BG vs. N vs. D vs. P vs. S vs. V) and 2 class (D vs. P) classifications, whereas the accuracy went up by 0.07%, 1.36%, and 5.18% for 5 class (BG vs. N vs. D vs. P vs. S), 4 class (D vs. P vs. S vs. V), and 3 class (D vs. P vs. S) classifications. For average cross-validation results, the accuracy went up by 0.97% for the 3 class classification; in contrast, the accuracy got down by 4.53%, 5.06%, 7.14%, and 7.25% for 6 class, 5 class, 4 class, and binary classifications. For external validation on Medetec dataset, we achieved an 8.56% improvement on specific fold accuracy, while the accuracy decreased by 6.93% for average cross-validation accuracy.

The comparison results discussed above show that the overall performance is down for the most complicated tasks using cross-validation. But considering the percentage decrement or increment, our developed model worked well considering the challenging factors of cross-validation. In cross-validation, there is no validation data to tune our model with compared to the holdout test method with a validation set. Also, cross-validation with a small number of samples is problematic as, for some folds, the training data may not contain enough diverse samples to train on, which was also reflected in the fold-wise accuracy variance. Nevertheless, we achieved good results for external validation considering the data difference among the AZH and Medetec datasets.

Finally, still with cross-validation on our hardest classifications, the WMC classifier outperforms the WIC and WLC classifiers, which again proves the power of multimodality and our developed WMC model. On the other hand, this cross-validation experiment shows the importance of having more data to build more robust and reliable deep learning models.

### Comparison with previous works

From Table 15, we can see that our work outperformed all the previous works by a good margin. As mentioned earlier, this comparison is not perfect as factors like dataset, model, training-validation-testing split, balance ness of the dataset, resources used for training, etc., are not the same as the previous works. But this comparison proves that multimodality using wound image and location can improve the wound classification results. We achieved a 7.5% improvement in accuracy for classifying Healthy Skin Vs. DFU Skin (N Vs. D) from Goyal et al.’s work^12^ on our AZH dataset. Compared to Aguirre et al.’s work^13^ of classifying VLU versus non-VLU (V vs. [N or D or P or S]) wounds, we achieved a significant 5.63% to 15% improvement in accuracy with the AZH dataset. In this experiment, we improved 5.63% for VLU vs. PU, 9.44% for VLU vs. DFU, 12.12% for VLU vs. Surgical, and 15% for VLU vs. Normal skin. Our developed classifier outperformed Alzubaidi et al.’s work^15^ on Normal Skin Vs. Abnormal (DFU) Skin (N vs. D) classification with 5.5% improvement in F1-score for the AZH experiment. Finally, compared to our previous work^16^, there are 13 similar experiments in our present work. We achieved a significant improvement with the multi-modal WMC network in all these experiments. In these 13 experiments, the accuracy improvement using WMC classifier from our previous work are: (1) 0.72% improvement in SV classification, (2) 0.1% improvement in DSV classification, (3) 6.16% improvement in BGNDV classification, (4) 5.9% improvement in BGNPV classification, (5) 1.96% improvement in BGNSV classification, (6) 8.94% improvement in BGNDP classification, (7) 0.32% improvement in BGNDS classification, (8) 1.59% improvement in BGNPS classification, (9) 4.7% improvement in BGNDPV classification, (10) 6.06% improvement in BGNDSV classification, (11) 1.65% improvement in BGNDPS classification, (12) 2.64% improvement in BGNPSV classification, and (13) 13.79% improvement in BGNDPSV classification. Both of these works have some pros and cons: in our previous work, we had a balanced dataset (all classes had the same no of images), where the current work has an unbalanced dataset (Table 2); the previous work used a very sophisticated ensemble classifier for image classification, where this work uses simple transfer learning with available DNN networks (VGG16, VGG19, etc.); the previous work only used wound images for training the classifier, where the current network uses both wound images and their corresponding locations for developing the classifier. Overall, this work outperforms all the previous works by a good difference.

### Limitations and scope of improvement

**Figure 9:**
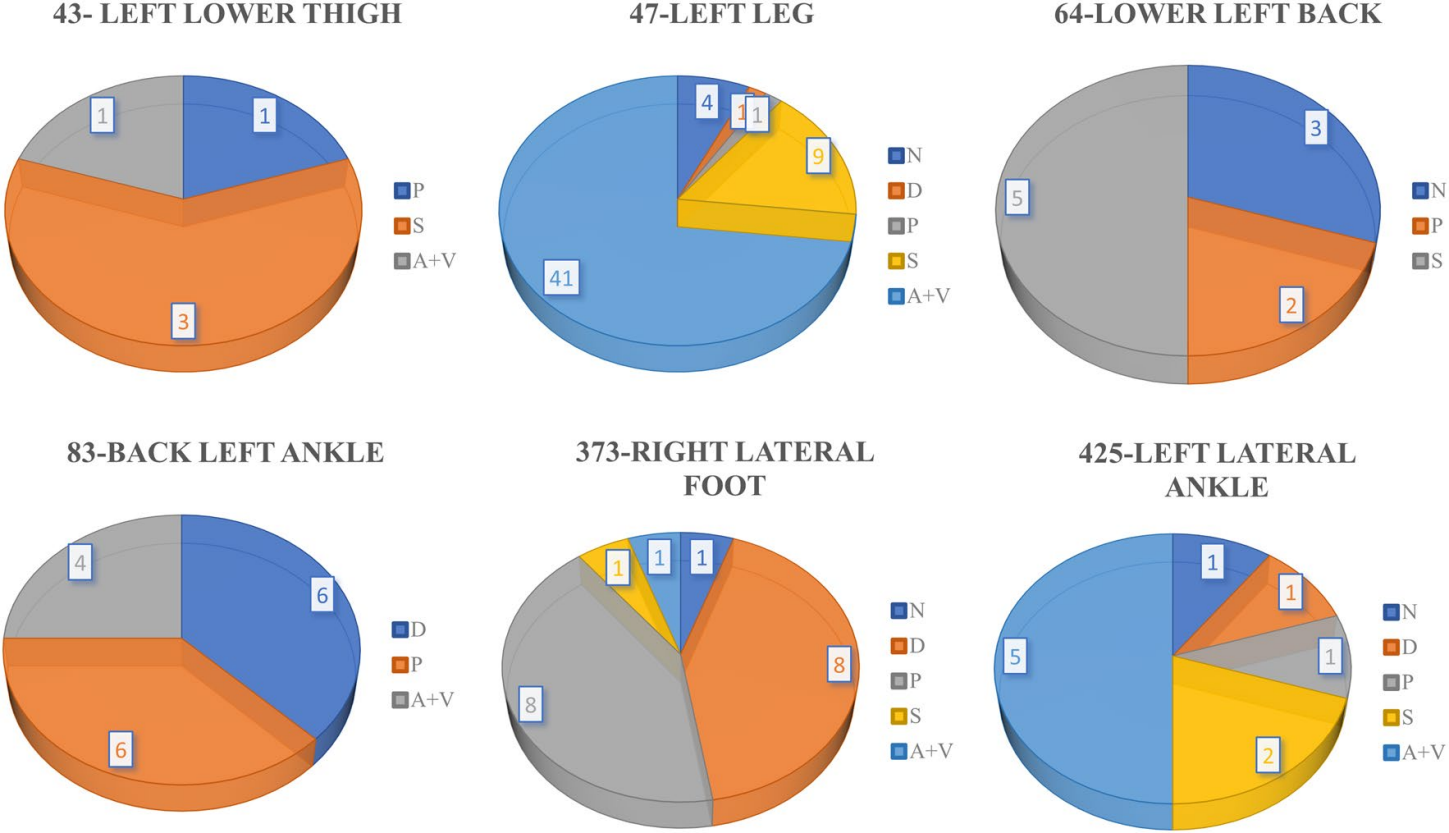
Examples of location overlaps on AZHMT dataset.

In Fig. 6, the WLC network’s performance is very poor compared to the WIC and WMC network. One important reason is that there were some overlaps among the normal (healthy) skin and other wound classes, as the normal skin is cropped from the wound images. In one patient’s wound image, a non-infected (normal) skin can be infected in another patient’s wound image, which produces these overlaps and thus decreases the WLC performance. Figure 7 shows that the WLC network’s performance was better than the WIC network as there is no normal skin (N) class in these classifications. The WLC network performance can be improved by increasing the number of data points, which can help increase the WMC network’s performance in the long run. Figure 9 shows some examples of location overlapping among different classes.

## Conclusion

This paper developed a multi-modal wound classifier (WMC) network using wound images and their corresponding locations to classify wounds into different classes. To the best of our knowledge, it is the first developed multi-modal network that uses images and locations for wound classification. This research is also the first work that classifies wounds according to their locations. We also developed a body map to help clinicians document the wound locations in the patient’s record to prepare the location data. The developed body map is currently used in the AZH wound center for location tagging to avoid inconsistency with location information. Three datasets with wound images and their corresponding locations were also developed and labeled by wound specialists of AZH wound center to perform many wound classification experiments. The multi-modal (WMC) network was created in the concatenation of two networks: wound image classifier (WIC) and wound location classifier (WMC). Developing the WIC network transfer learning was used with top-rated deep learning models. The WLC network was also developed using deep learning models that are popular for controlling categorical data. A large number of experiments with a range of binary to six-class classifications were performed in three datasets, where many wound classifications were never performed before, to the best of our knowledge. The results produced by the WMC network were much better than the results produced from the WIC or WLC networks, and these results beat all the previous experimental results. In future experiments, the performance of the WMC network can be improved further by using more specific WIC and WLC networks for wound image classifications and wound location classifications, respectively. There are some overlaps in the wound location data, for which the WLC network produced lower accuracy compared to WIC and WMC networks. Increasing the number of data can improve the location (WLC) classifier. We are planning to add more modalities (pain, palpation findings, general findings, area, volume, age, sex, BMI, etc.) in our future works. Overall, the developed WMC classifier can significantly speed up the automation of wound healing systems in the near future.

Deep learning-based wound care algorithms can improve patient outcomes with higher efficiency and lower costs. Accurate classification of wound types can help clinicians diagnose wound problems more quickly and find proper treatment plans. AI wound analysis equipped with mobile devices would reduce the burden of wound care providers and allow rapid diagnosis and quality treatment, especially for rural regions with much less accessible resources. With the development of these models, clinicians in resource-limited settings can quickly identify the types of wounds and seek help from experts accordingly based on the initial wound assessment. This pipeline improves diagnosis efficiency and accuracy simultaneously. The major limitation of the proposed methods is the data scarcity to improve the model generality and give both patients and physicians proper technical training to use these developed deep learning-based applications.

## Data Availability

The AZH dataset is currently available at https://github.com/uwm-bigdata/Multi-modal-wound-classification-using-images-and-locations. Unfortunately, due to authorship conflict, we cannot make the Medetec and AZHMT datasets public.
